# The Sex Chromosome Trisomy mouse model of XXY and XYY: metabolism and motor performance

**DOI:** 10.1186/2042-6410-4-15

**Published:** 2013-08-08

**Authors:** Xuqi Chen, Shayna M Williams-Burris, Rebecca McClusky, Tuck C Ngun, Negar Ghahramani, Hayk Barseghyan, Karen Reue, Eric Vilain, Arthur P Arnold

**Affiliations:** 1 Department of Integrative Biology & Physiology, University of California, Los Angeles, 405 Hilgard Avenue, Los Angeles, CA 90095, USA; 2 Department of Human Genetics, David Geffen School of Medicine at UCLA, University of California, Los Angeles, 405 Hilgard Avenue, Los Angeles, CA 90095, USA; 3 Laboratory of Neuroendocrinology of the Brain Research Institute, University of California, Los Angeles, 405 Hilgard Avenue, Los Angeles, CA 90095, USA; 4 Department of Medicine, David Geffen School of Medicine at UCLA, University of California, Los Angeles, 405 Hilgard Avenue, Los Angeles, CA 90095, USA; 5 Departments of Pediatrics and Urology, David Geffen School of Medicine at UCLA, University of California, Los Angeles, 405 Hilgard Avenue, Los Angeles, CA 90095, USA

**Keywords:** Klinefelter, Sex chromosome trisomy, XXY, XYY, Mouse, X chromosome, Y chromosome, Body weight, Obesity

## Abstract

**Background:**

Klinefelter syndrome (KS), caused by XXY karyotype, is characterized by low testosterone, infertility, cognitive deficits, and increased prevalence of health problems including obesity and diabetes. It has been difficult to separate direct genetic effects from hormonal effects in human studies or in mouse models of KS because low testosterone levels are confounded with sex chromosome complement.

**Methods:**

In this study, we present the Sex Chromosome Trisomy (SCT) mouse model that produces XXY, XYY, XY, and XX mice in the same litters, each genotype with either testes or ovaries. The independence of sex chromosome complement and gonadal type allows for improved recognition of sex chromosome effects that are not dependent on levels of gonadal hormones. All mice were gonadectomized and treated with testosterone for 3 weeks. Body weight, body composition, and motor function were measured.

**Results:**

Before hormonal manipulation, XXY mice of both sexes had significantly greater body weight and relative fat mass compared to XY mice. After gonadectomy and testosterone replacement, XXY mice (both sexes) still had significantly greater body weight and relative fat mass, but less relative lean mass compared to XY mice. Liver, gonadal fat pad, and inguinal fat pad weights were also higher in XXY mice, independent of gonadal sex. In several of these measures, XX mice also differed from XY mice, and gonadal males and females differed significantly on almost every metabolic measure. The sex chromosome effects (except for testis size) were also seen in gonadally female mice before and after ovariectomy and testosterone treatment, indicating that they do not reflect group differences in levels of testicular secretions. XYY mice were similar to XY mice on body weight and metabolic variables but performed worse on motor tasks compared to other groups.

**Conclusions:**

We find that the new SCT mouse model for XXY and XYY recapitulates features found in humans with these aneuploidies. We illustrate that this model has significant promise for unveiling the role of genetic effects compared to hormonal effects in these syndromes, because many phenotypes are different in XXY vs. XY gonadal female mice which have never been exposed to testicular secretions.

## Background

Klinefelter syndrome (KS), caused by the karyotype XXY, is characterized by small testes, azoospermia, low testosterone, hypergonadotropism, and gynecomastia [1,2]. The prevalence of the KS karyotype is 1 in 600 live male births, making it the most common genetic cause of male infertility [3-6]. In addition to the gonadal phenotypes, KS men have non-gonadal traits and symptoms. For example, KS men are taller than XY siblings or peers and exhibit specific cognitive deficits—especially involving language, social, and executive functioning skills [7-16]. Another sex chromosome trisomy, XYY, does not cause infertility or hypogonadism, but XYY boys exhibit similar cognitive deficits to those seen in KS [9,15].

KS men also experience an increased prevalence of several health problems [17,18], some of which are usually more common in women than in men including breast cancer [19,20], osteoporosis [21,22], and autoimmune diseases such as rheumatoid arthritis and systemic lupus erythematosus [23-26]. Relative to XY males, KS men have increased body fat, specifically abdominal fat, as well as increased rates of hyperinsulinemia, insulin resistance, type II diabetes, and metabolic syndrome [27-33]. It is generally believed that hypogonadism is primarily responsible for the metabolic phenotype seen in KS patients. Treatment with testosterone can provide improved outcomes for some KS phenotypes [34,35] but is not sufficient to improve metabolic features [28]. Moreover, KS boys suffer from metabolic syndrome before puberty, when testosterone levels are similarly low in both KS boys and controls [31]. Additionally, a recent study comparing men with KS vs. men with idiopathic hypogonadotrophic hypogonadism (IHH) showed that men with KS had greater rates of diabetes than men with IHH both before and after both groups received testosterone replacement therapy [33]. Taken together, these results suggest that there are likely to be direct genetic effects of the second X chromosome on metabolism, not exclusively mediated by reduced androgen levels in KS males.

Boys and men with KS also have impaired motor cognition and function. Several studies suggest that KS boys are clumsy and do not like sports [14]. KS boys show impairments in gross motor function [36-38], fine motor function [14,36,39], sensorimotor integration [37,38], and overall strength and speed [38]. These deficits begin early in life [37] and persist into adulthood [40,41]. Although XYY males are studied less often, they also show motor deficits similar to those seen in KS including fine motor impairment, gross motor impairment, decreased sensorimotor integration, and deficits in coordination [37,38].

KS males may experience lower levels of testicular androgens at various life stages. These hormones are known to act pre- and post-natally to cause permanent (‘organizational’) effects on various tissues. They also act more acutely and reversibly at all life stages (‘activational’ effects) [42]. Although it is impossible to cleanly separate direct genetic effects from those caused by lower hormone levels in humans, here, we offer a new mouse model of XXY and XYY with improved discrimination of hormonal vs. chromosomal effects, as well as their interactions.

The Sex Chromosome Trisomy (SCT) model produces XXY, XYY, XY, and XX mice, each genotype with either testes or ovaries, all within the same litters. The model allows comparison of four different sex chromosome genotypes and models both XXY and XYY human trisomies. The effects of sex chromosome complement are tested after exposure to ovarian or testicular secretions and after removal of these hormones by adult gonadectomy, which allows for assessment of the hormonal independence of sex chromosome effects as well as assessment of interactions of sex chromosomes and hormones. To validate the SCT model, we measured body composition and motor function, which are each known to be significantly different in KS men relative to XY males.

## Methods

### Mice

**Figure 1:**
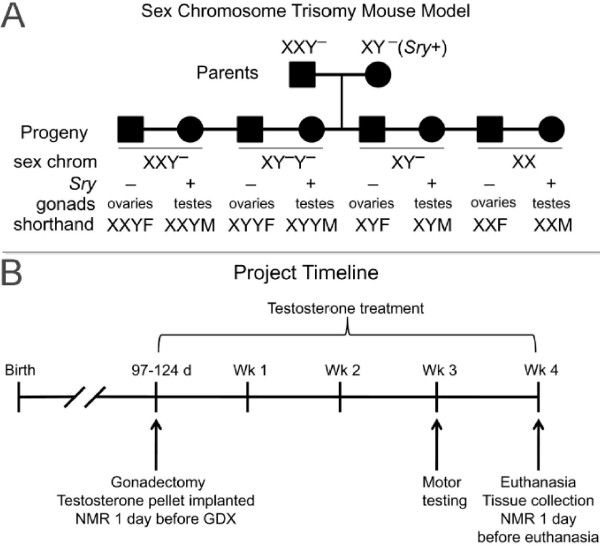
**Cross producing the eight genotypes of the SCT model (A) and project timeline (B).** All mice were outbred MF1 strain. We attempt to maintain genetic diversity in our colony, but the X chromosomes of all mice used here were identical and derived originally from mating an XY mouse with his XO dam to produce a line of mice with identical X chromosomes bred onto outbred stock (P Burgoyne, personal communication). Thus, in these experiments, differences between mice with one vs. two X chromosomes were attributable to the number of X chromosomes, not to a difference in X alleles.

The Sex Chromosome Trisomy model (designed by P Burgoyne, personal communication; similar to mice used by Park et al. [43]) involves mating XXY^−^ females with the XY^−^(*Sry+*) males (Figure 1A). The Y^−^ (‘Y minus’) chromosome is deleted for the testis-determining gene *Sry* so that the Y chromosome no longer causes differentiation of testes [44]. The XY^−^(*Sry+*) fathers possess an *Sry* transgene inserted into an autosome, which complements the lack of *Sry* on the Y chromosome and produces a fully functional male [45-47]. The mothers produce X and XY^−^ eggs, and the fathers produce X or Y^−^ sperm each with or without *Sry*. A total of eight genotypes are produced: gonadal males XXY^−^(*Sry+*), XY^−^Y^−^(*Sry+*), XY^−^(*Sry+*), and XX(*Sry+*), as well as gonadal females XXY^−^, XY^−^Y^−^, XY^−^, and XX (Figure 1A). Here, we refer to these genotypes respectively as XXYM, XYYM, XYM, XXM, XXYF, XYYF, XYF, and XXF. For this study, the XXY^−^ mothers were produced by crossing XX females to XY^−^Y*^X^(*Sry+*) males, which are made by crossing XY*^X^ females to XY^−^(*Sry+*) males (see [48-51] for discussion of the Y*^X^ chromosome). More recently, we produce XXY^−^ mothers by breeding XY^−^ females to wild-type XY males, a more straightforward breeding scheme in which about 30% of female offspring are XXY^−^. To our knowledge, the SCT model is easier to produce using outbred rather than inbred strains. Group sizes were 11 XXYF, 9 XXYM, 8 XYYF, 8 XYYM, 21 XYF, 14 XYM, 14 XXF, and 9 XXM. Measurements of brain gene expression and partner preference behavior of these mice are reported (TC Ngun et al., unpublished work).

All experiments were approved by the UCLA Animal Research Committee. Mice were group-housed based on their gonadal sex and maintained at 23°C on a 12:12 light/dark cycle. Mice were fed regular chow diet with 5% fat (LabDiet 5001, St. Louis, MO, USA).

### Gonadectomy and hormonal implants

The SCT model primarily models XXY and XYY male genotypes of humans, which are normally only present in individuals with testes. Accordingly, the effects of these genotypes, relative to normal XY genotype, might be detected only when testosterone is present. Therefore, in the current study, all mice were gonadectomized during adulthood at 97–124 days of age and implanted with a testosterone pellet at the time of gonadectomy (Figure 1B). The intent was to make circulating gonadal hormone levels as similar as possible so that group differences could not be attributed to different levels of gonadal hormones in adulthood. Testosterone pellets contained 5 mm of testosterone (Steraloids, Inc., Newport, RI, USA) packed into a Silastic tube (1.57 mm ID × 2.41 mm OD, Dow Corning, Midland, MI, USA) and sealed at each end with 3 mm of medical grade Silastic adhesive (Dow Corning). Age of the mice at the time of gonadectomy did not differ across groups (overall ANOVA, *p* > 0.05).

### Genotyping

Karyotypes were determined from metaphase spreads prepared from tail or ear fibroblasts, using routine methods [52].

### Measurement of plasma levels of testosterone

Plasma was collected from the carotid artery following decapitation and then stored at −20°C until radioimmunoassay was performed by the Ligand Assay and Analysis Core at the University of Virginia Center for Research in Reproduction (supported by NICHD (SCCPIR) grant U54-HD28934). Measurements were performed in singlet reactions using Siemens Medical Solutions Diagnostics' testosterone RIA (Malvern, PA, USA) with a reportable range of 0.72–111.00 ng/L.

### Body weight, body composition, and metabolic measures

Body weight and composition were measured just prior to gonadectomy and at the end of the experiment (see Figure 1B). A nuclear magnetic resonance (NMR) Mouse Minispec apparatus (Bruker Woodlands, TX, USA) with Echo Medical Systems (Houston, TX, USA) software was used to measure fat and lean mass [53] with coefficients of variation of less than 3%. Correlation between NMR and gravimetric measurements is better than 0.99. NMR was performed the day before gonadectomy (GDX) and 3 weeks after GDX.

At the end of the experiment (Figure 1B), the weights of two specific fat pads were measured, visceral fat of the peri-gonadal depot (referred to here as gonadal fat) and the subcutaneous fat depot from the femoral region (referred to as inguinal fat). Both depots were assessed because visceral and subcutaneous fat depots are differentially regulated and have diverse positive and negative effects on metabolic syndrome in humans and mice [54,55].

### Testis size

At the time of gonadectomy, testes were classified as small, medium, or normal (large) by a researcher who was blind to the genotype. All XYM had normal testes, all XYYM had medium testes, and all XXM and XXYM had small testes, except for one XXYM that had medium testes.

### Motor tests 3 weeks after GDX

At 18–22 days after GDX, motor function was assessed using two sensitive tests, the challenging beam traversal test and the pole test [56,57]. The challenging beam test required mice to traverse a 1-m-long beam made up of four 25-cm-long sections of decreasing width (3.5 cm at its widest and 0.5 cm at its narrowest, decreasing by 1-cm increments). Mice were trained to traverse the beam starting at the widest section and ending at their home cage, which was placed at the end of the narrowest section. Mice received two consecutive days of training, making five traversals each day, and were tested on the third day. On the testing day, the beam traversal was made more challenging by adding a wire grid to the top of the beam so that mice had to grasp the wire to complete traversal, and errors could be calculated based on misses or slips while grasping. During testing, mice were video recorded making five traversals of the beam. The following variables were scored from videos of the behavior: time to traverse the beam, number of steps, and number of errors.

The pole test involved placing the mouse head up at the top of a 50-cm-long, 1-cm-diameter vertical wooden pole with the base of the pole in the animal's home cage. Mice were trained to turn around, to face down, and traverse down the pole into their home cage. Mice received two consecutive days of training, making five traversals each day. On the third day, the mice were video recorded making five traversals. The following variables were later scored from videos: time to turn around and time to reach the base of the pole.

Training for the challenging beam traversal test and the pole test were done consecutively for each mouse, such that each mouse completed training for the pole before training for the beam in a single session. Testing on the third day was done similarly, with each mouse completing the pole test then immediately completing the beam test.

### Statistical analyses

An overall analysis of variance (ANOVA) was used to compare the eight groups, with factors of gonadal sex (*Sry* present vs. absent) and genotype (four levels). In addition, our *a priori* hypotheses were that XY mice differed pairwise with XX, XXY, and XYY, and these comparisons were tested with three separate two-way ANOVAs with factors of gonadal sex and genotype (two levels). In two cases, we used body weight as a covariate in an analysis of covariance (ANCOVA) to determine if group differences in the dependent variable were attributable to differences in body weight.

## Results

### In gonadally intact mice, XXY (but not XYY) sex chromosome complement was associated with greater body weight and adiposity

**Figure 2:**
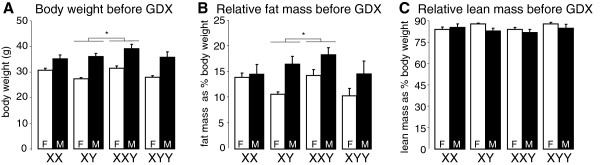
**Body weight and composition just before gonadectomy. (A)** Gonadal males were about 25% heavier than gonadal females (*F*(1,86) = 42.09, *p < 0.000001, overall ANOVA). In pairwise tests, XY mice weighed 13% less than XXY regardless of gonadal type (*F*(1,51) = 6.65, *p = 0.013). **(B)** Gonadal males had about 32% more body fat as percentage of body weight compared to gonadal females (*F*(1,86) = 6.05, *p = 0.015, overall ANOVA). In pairwise comparisons, XY had about 24% less body fat as percentage of body weight than XXY irrespective of gonadal sex (*F*(1,51) = 4.74, *p = 0.034). **(C)** Lean mass as percentage of body weight was unaffected by sex or sex chromosome complement. *F* gonadal females, *M* gonadal male. Values are mean ± SEM.

Mice were weighed just before gonadectomy. At this time, gonadal males were significantly heavier (25%) than gonadal females regardless of sex chromosome complement, and there was no overall effect of sex chromosome complement (Figure 2A). In pairwise comparisons, XXY mice weighed significantly more (13%) than XY mice regardless of gonadal type, but XY did not differ from XX or XYY. Gonadal males also had significantly greater relative fat mass (32%, Figure 2B), and significantly greater (70%) absolute fat mass than gonadal females (in Additional file 1: Figure S1A), but there was no overall effect of sex chromosome complement on either measure. In pairwise comparisons, XXY mice had significantly more (24%) relative fat mass than XY mice, but XY did not differ from XX or XYY, and there was no effect of sex chromosome complement on absolute fat mass. Relative lean mass was not affected by sex or sex chromosome complement in the overall analysis (Figure 2C), but gonadal males had significantly greater (21%) absolute lean mass than gonadal females (in Additional file 1: Figure S1B); there was a significant effect of sex chromosome complement on absolute lean mass. In pairwise comparisons for relative lean mass, there was no significant effect of sex chromosome complement, but XXY mice had significantly more (10%) absolute lean mass than XY (in Additional file 1: Figure S1B), but XY did not differ from XX or XYY. Thus, the gonadally intact XXY mice resembled KS men in having increased body weight compared to XY mice, which was caused, in part, by increased proportional fat mass. These differences were independent of gonadal sex and suggest that XXY chromosome complement influences metabolism leading to altered body composition.

### After GDX and testosterone treatment, XXY mice had greater body weight and adiposity than XY mice

**Figure 3:**
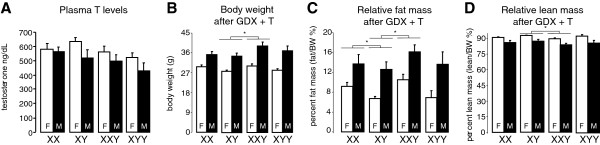
**Plasma testosterone and body weight and composition after gonadectomy and testosterone treatment. (A)** Plasma testosterone did not differ as a function of sex chromosome complement within sex, but gonadal females had overall 14% higher levels of testosterone (*F*(1,84) = 4.91, *p* = 0.029). **(B)** Gonadal males were about 26% heavier than gonadal females (*F*(1,84) = 63.13, *p < 0.000001), and XY mice weighed 13% less than XXY irrespective of gonadal sex (*F*(1,51) = 7.40, *p = 0.009). **(C)** Gonadal males had 69% more relative fat mass than gonadal females (*F*(1,84) = 37.75, *p < 0.000001), and there was a significant overall effect of sex chromosome complement (*F*(3,84) = 4.30, *p = 0.007). XY mice had 43% less relative fat mass than XXY mice (*F*(1,51) = 13.18, *p < 0.0007), and 21% less than XX mice (*F*(1,53) = 4.20, *p = 0.045), irrespective of gonadal sex. **(D)** Relative lean mass was 6% lower in gonadal males than in females (*F*(1,84) = 29.21, *p = 0.000001, overall ANOVA). In pairwise comparisons, XY had 4% greater relative lean mass than XXY (*F*(1,51) = 6.63, *p = 0.013). Values are mean ± SEM.

To remove confounding effects of endogenous gonadal secretions, we performed GDX and restored testosterone in mice of all genotypes by testosterone pellet implantation. Three weeks after GDX and testosterone pellet implantation, all mice had testosterone levels in the physiological range (170–1,440 ng/dL). There were no differences across genotypes, but gonadal females had significantly higher (14%) testosterone levels than males (Figure 3A). The sex difference in the level of testosterone is possibly explained by the larger body size of males, because using body weight as a covariate in an ANCOVA eliminated the significant sex difference in the level of testosterone. Thus, we generated mice that do not differ in testosterone levels across genotype within sex. We assessed body weight and composition and related metabolic parameters in these animals.

Three weeks after GDX and testosterone pellet implantation, gonadal males were still significantly heavier (26%) than gonadal females (Figure 3B), but there was no overall effect of sex chromosome complement. In pairwise comparisons, XXY mice weighed 13% more than XY, but XY did not differ from XX or XYY. Gonadal males had significantly greater relative (69%) and absolute (121%) fat mass than gonadal females (Figure 2C; in Additional file 1: Figure S2A). In addition, sex chromosome complement had a significant overall effect on relative fat mass. In pairwise comparisons, XXY mice had 43% more relative fat mass than XY mice, and XX had 21% more than XY mice (Figure 3C). Absolute fat mass was 60% higher in XXY compared to XY mice (in Additional file 1: Figure S2B). Fat mass was not different in XY relative to XX or XYY. Relative lean mass was 6% lower in gonadal males than in gonadal females (Figure 3D), but absolute lean mass was 17% greater in gonadal males than females (in Additional file 1: Figure S2B). In pairwise comparisons, XXY had 4% lower relative lean mass than XY, and XYY had 7% more absolute lean mass than XY. XY did not differ from XX or XYY in relative or absolute lean mass. Thus, the XXY genotype (but not XYY) was associated with increased body weight due to increases in both absolute fat and lean mass, and these animals had a higher proportion of their body mass as fat than XY mice.

### GDX and testosterone treatment interact with sex and genotype to change body composition

**Figure 4:**
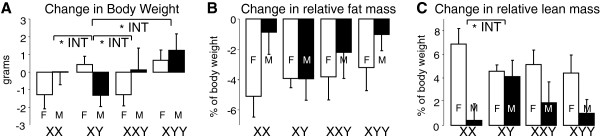
**Change in body weight and composition caused by gonadectomy and testosterone treatment. (A)** XYY mice gained more weight than XY (*F*(1,46) = 4.58, *p* = 0.038, main effect). In addition, the change in body weight in males vs. females depended on sex chromosome complement when comparing XY with XX (*F*(1,53) = 0.020, *p* = 0.02, *INT) and XY with XXY (*F*(1,51) = 5.42, *p* = 0.024, *INT). **(B)** Gonadal females overall lost more relative fat mass than gonadal males (*F*(1,84) = 4.37, *p* = 0.039), but there was no significant effect of sex chromosome complement. **(C)** Gonadal females overall gained more relative lean mass than gonadal males (*F*(1,84) = 13.82, *p* = 0.0004). The difference between XY and XX was different depending on their gonadal sex (*F*(1,53) = 9.56, *p* = 0.003, *INT). *INT indicates significant interactions of gonadal sex and sex chromosome complement. Values are mean ± SEM.

To assess group differences in the effect of gonadectomy plus testosterone treatment, we used the change in body weight and composition (measurements at the end of the experiment minus measurements before GDX) as the dependent variable. There was no significant overall effect of sex or sex chromosome complement on the change in body weight. In pairwise comparisons, XYY gained more weight than XY (Figure 4A). Females had a significantly greater loss in relative fat mass (Figure 4B) and greater gain in relative lean mass (Figure 4C) compared to males, but there was no overall effect of sex chromosome complement on these measures. There were significant interactions between sex chromosome complement and gonadal sex in pairwise comparisons of XY with all three other groups for body weight and when comparing XY with XX for change in relative lean mass (Figure 4A,C). There were no significant effects of sex or sex chromosome complement on the change in absolute fat mass or lean mass (data not shown).

### After GDX and testosterone treatment, XXY mice had heavier metabolic tissues than XY mice

**Figure 5:**
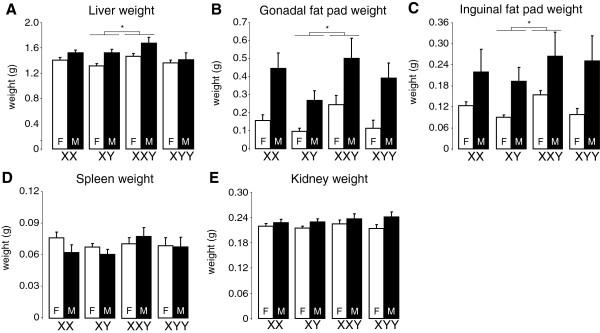
**Tissue weights after gonadectomy and treatment with testosterone.** All tissue weights except spleen were greater in gonadal males than females in the overall ANOVA (liver, *F*(1,85) = 13.05, *p = 0.0005; inguinal fat, *F*(1,86) = 19.41, *p = 0.00003; gonadal fat, *F*(1,50) = 23.29, *p = 0.00001; kidney, *F*(1,86) = 7.28, *p = 0.008), and sex chromosome complement affected liver weight in the overall ANOVA (*F*(3,85) = 3.84, *p = 0.012). XXY mice had heavier livers **(A)**, and gonadal **(B)** and inguinal **(C)** fat pads, compared to XY (liver, *F*(1,51) = 8.13, *p = 0.006; gonadal fat *F*(1,29) = 7.61, *p = 0.01; inguinal fat, *F*(1,51) = 4.55, *p = 0.038). There were no group differences in spleen **(D)** weights. Values are mean ± SEM.

To further define the basis for differences in body weight and composition among genotypes, we measured the weights of key metabolic tissues, including representative visceral and subcutaneous fat pads (gonadal and inguinal fat depots, respectively), liver, spleen, and kidney. Except for the spleen, each of these tissues was significantly heavier in gonadal males than females (Figure 5). There was also a significant overall effect of sex chromosome complement on liver weight. In pairwise comparisons, XXY had significantly heavier liver, gonadal fat pad and inguinal fat pad compared to XY (Figure 5A–C), and XY did not differ from XX or XYY. There were no group differences in spleen weights.

### Differences in body weight and composition between XXY and XY occurred in gonadal female groups

Because XXY male mice may have lower testosterone levels than XY males during development [58,59], differences between XXY and XY mice could be caused by organizational effects of testicular hormones. To test whether differences exist under conditions in which the XXY vs. XY comparison is unlikely to be confounded by androgen levels in this manner, we assessed sex chromosome effects in females only, using one-way ANOVAs with sex chromosome complement as the factor. Before GDX (Figure 2; in Additional file 1: Figure S1), there was a significant effect of sex chromosome complement in females on body weight (*F*(3,50) = 4.78, *p* = 0.005, overall one-way ANOVA), relative fat mass (*F*(3,50) = 2.79, *p* = 0.05), absolute fat mass (*F*(3,50) = 3.39, *p* = 0.025), relative lean mass (*F*(3,50) = 3.73, *p* = 0.017), and absolute lean mass (*F*(3,50) = 3.25, *p* = 0.029). Notably, in pairwise comparisons, XXYF differed from XYF in each of these measures (with greater body weight and fat and less relative lean mass, *p* < 0.009, *t* test). XYF also differed from XXF in each of these measures except relative fat mass (*p* < 0.045, *t* test).

After GDX and testosterone treatment (Figure 3; in Additional file 1: Figure S2), there was a significant effect of sex chromosome complement on body weight (*F*(3,48) = 2.82, *p* = 0.049, overall one-way ANOVA), relative fat mass (*F*(3,48) = 5.27, *p* = 0.003), absolute fat mass (*F*(3,48) = 5.66, *p* = 0.002), and relative lean mass (*F*(3,48) = 4.42, *p* = 0.008). In pairwise comparisons, XXYF differed from XYF in each of these measures (with greater body weight and relative fat and less relative lean mass, *p* < 0.022, *t* test). XYF also differed from XXF in each of these measures (*p* < 0.036, *t* test). In response to GDX and testosterone treatment, there was a significant effect of sex chromosome complement on the change in body weight (*F*(3,48) = 2.81, *p* = 0.049, overall one-way ANOVA) (Figure 4A), but not in other measures. In pairwise comparisons, XXYF had less change in body weight than XYF (*p* = 0.026, *t* test), and XYF had greater change in body weight than XXF (*p* = 0.044, *t* test) (Figure 4A). In tissue weights (Figure 5), there was a significant effect of sex chromosome complement for inguinal fat pad weight (*F*(3,50) = 7.67, *p* = 0.0003, overall one-way ANOVA) and relative inguinal fat pad weight (*F*(3,50) = 7.58, *p* = 0.0003), but not for other tissues. In pairwise comparisons, XXYF differed from XYF for liver weight (*p* = 0.012, *t* test), gonadal fat pad weight (*p* = 0.019, *t* test), and inguinal fat pad weight (*p* < 0.000001, *t* test) (Figure 5). XYF also differed from XXF for inguinal fat pad weight (*p* = 0.007, *t* test). There were no significant differences between XYF and XYYF on any measure. Thus, even under conditions in which mice have never had testes, the second X chromosome of the XXY genotype influences body composition and metabolic tissue weights.

### XYY mice differed from XY mice in tests of motor function

**Figure 6:**
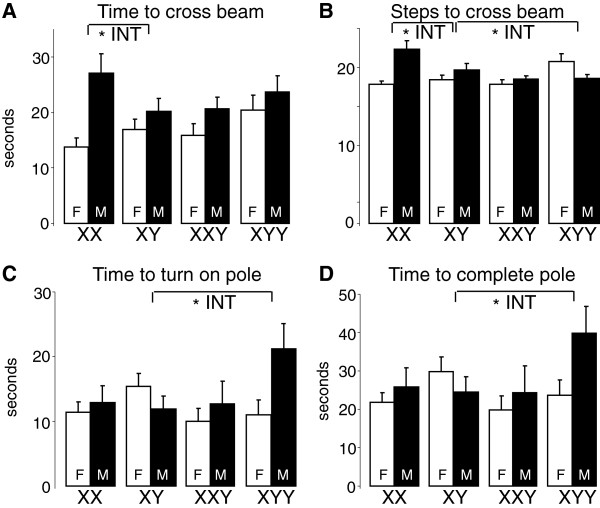
**Motor performance. (A)** In the challenging beam test, gonadal males took 38% longer to cross the beam compared to females (*F*(1,85) = 12.45, *p* = 0.0007, overall ANOVA) XX gonadal males took longer to cross the beam than XY males, but XX and XY females did not differ (*F*(1,53) = 4.60, *p* = 0.037, *INT). **(B)** Gonadal males took 7% more steps to cross the beam compared to gonadal females (*F*(1,85) = 4.41, *p* = 0.04, overall ANOVA), but the effect of sex depended on the genotype (*F*(3,85) = 7.02, *p* = 0.0003, *INT). In pairwise comparisons, the difference between XY and XX, and XY and XYY, depended on sex (*F*(1,53) = 5.25, *p* = 0.026 and *F*(1,46) = 4.78, *p* = 0.034, respectively, *INT). **(C)** In time to turn on the pole, the difference between XY and XYY mice depended on their sex (*F*(1,46) = 6.80, *p* = 0.012, *INT). **(D)** In time to complete the pole test, the difference between XY and XYY mice depended on their sex (*F*(1,46) = 4.62, *p* = 0.037, *INT). *INT indicates significant interactions of gonadal sex and sex chromosome complement. Values are mean ± SEM.

In the challenging beam test, gonadal males took significantly longer (38%) to cross the beam compared to females (Figure 6A). In pairwise comparisons, there was a significant sex by sex chromosome complement interaction for XY compared to XX such that XX gonadal males (but not gonadal females) took longer to cross the beam than XY. Gonadal males also took significantly more steps to cross the beam compared to gonadal females (Figure 6B), and the effect of sex differed depending on sex chromosome complement. In pairwise comparisons, there were sex by sex chromosome complement interactions such that XX took more steps than XY but only in gonadal males, and the XYY vs. XY difference was not consistently in the same direction for gonadal males and females. There were no significant sex or sex chromosome complement effects on errors in any section of the beam (data not shown).

For the pole test, there was no overall effect of sex or sex chromosome complement (Figure 6C,D), but in pairwise comparisons, there were significant sex by sex chromosome complement interactions such that XYY males (but not females) took longer to turn and complete the test than XY. Because the mice differed in body weight and because increased body weight could make completion of the pole test more difficult, we analyzed the pole data using an ANCOVA with weight as the covariate but found that weight does not explain any of the group differences.

## Discussion

Here we introduce a novel mouse model, the Sex Chromosome Trisomy model, which compares XXY, XYY, XY, and XX mice within litters. Because gonadal sex is independent of sex chromosome complement, the effects of sex chromosome complement can be assessed independently of the exposure to testicular hormones. We find that XY and XXY mice differ in several measures of body composition and metabolic tissues including body weight, fat mass, lean mass, and metabolic tissue weights. Notably, these differences exist in XXY vs. XY gonadal females as well as in gonadal males, suggesting that these differences are caused, at least in part, by direct genetic effects of the second X chromosome acting outside of the gonads rather than exclusively by lower levels of testicular androgens in XXY mice. The results represent an initial validation of the SCT model, because XXY mice differ from XY mice in several phenotypes that parallel the features of KS and because the model unveils sex chromosome effects that would be difficult to study in humans or in previously described mouse models.

KS men have increased rates of obesity, especially abdominal obesity, and increased rates of type II diabetes [27-33]. In this study, we show that XXY mice mimic the KS phenotype seen in humans because they also have increased body weight, increased body fat, and increased abdominal fat pad weight, relative to XY. Also similar to KS patients, the XXY mice differ in these measures both before and after manipulations of testosterone levels. Importantly, these group differences were seen in both gonadal male and female XXY mice, implicating direct genetic effects rather than hormone effects in these traits. These results supports a growing body of human literature that suggests that low testosterone levels do not explain the higher rates of metabolic disease in KS patients and that testosterone treatment is not useful in preventing metabolic disease in these patients [28,31,33].

We hypothesized that XXY and XYY mice would have motor deficits compared to XY mice as seen in human patients with similar sex chromosome complement [37,38]. We found that XXY gonadal males did not perform differently from XY males on two tests of motor function but rather that the XYY males performed worse on the pole test. The XYY vs. XY differences were not found in gonadal females, suggesting that they represent the effects of an overdose of Y genes that require testicular hormones. For example, the overdose of Y genes may act on mechanisms that are masculinized by testosterone before adulthood. These motor impairments may model those seen in XYY humans, such as impairments in gross motor function and coordination [37,38]. Our failure to detect motor impairments in XXY mice suggests either that XXY mice do not effectively model the motor deficits found in humans or that we used tests that were not sensitive measures of the specific type of motor impairment seen in KS, such as fine motor dexterity.

The differences between XXY and XY mice that we report could be the result of several genetic mechanisms that operate differently in mice with two vs. one X chromosome [60]. About 3% of mouse X genes escape inactivation and are expressed from both X chromosomes [61]. Among these are six genes (*Ddx3x*, *Eif2s3x*, *Kdm5c*, *Kdm6a*, *Uba1*, and *Usp9x*) that are consistently reported to be expressed higher in one or more of the following patterns: XX > XO, XX > XY, and XXY > XY, in numerous mouse tissues including the brain [62-67]. These six genes are X-linked in both humans and mice, and most of them escape X inactivation in both species [61,68-71]. We consider these genes as top candidates for differences between XXY and XY groups found here. Indeed, expression profiling from the mice used in the present study confirmed higher expression of two of these genes in XXY vs. XY (*Kdm6a* and *Eif2s3x*) (TC Ngun et al., unpublished work). A second category of candidate genes are those that receive a parental imprint, because of the presence of both parental imprints in XXY mice, relative to the exclusively maternal imprint in XY. This class of genes is not well described but may include *Xlr3b*[72] and others [73,74]. Although XXY karyotype in humans sometimes results from maternal non-disjunction of X genes, in which case both X chromosomes would have a maternal imprint, the SCT model produces only XXY mice with one X chromosome from each parent, a condition found also in some KS patients. A third category is more hypothetical and includes genes that may be regulated indirectly by heterochromatizing factors that could be modulated by the presence of a large inactive X chromosome in cells with two X chromosomes [75,76].

The findings that sex chromosome complement is a significant contributing factor to body weight and body composition is not unexpected considering that our previous work on the Four Core Genotypes mouse model has shown that there are large sex chromosome effects in mice such that mice with two X chromosomes are heavier than mice with one X chromosome, regardless of gonadal sex, in both C57BL/6J and MF1 backgrounds [48,67]. In addition, in MF1 mice, a second sex chromosome of either type, X or Y, increases body weight and adiposity, relative to mice with one sex chromosome. In these previous reports, the sex chromosome effects were particularly large several months after removal of the gonads in adulthood. In the present study, we have also found similar sex chromosome effects both before and after adult gonadectomy with simultaneous treatment with testosterone. Unexpectedly, although the hormone treatments resulted in the same levels of testosterone in gonadal males and the same levels in gonadal females, the levels were higher in females than males. The sex difference in hormone levels are probably related to body size, such that equal-sized capsules produced higher hormone levels in the females, which were smaller. This inference is supported by the finding that the sex difference in testosterone levels was not significant when body weight was used as a covariate in an ANCOVA. Thus, the sex difference in level of testosterone at the end of the study could have contributed to the male-female differences found in some of the traits.

Several previous mouse models have been used to model KS [77-79]. One mouse model involves a four-generation breeding scheme that produces C57BL/6 XXY and XY mice in the same litters [51-53]. This model has been used fruitfully to discover important phenotypes associated with the XXY genotype in mice (e.g., hypogonadism, osteoporosis, and learning and social behavioral phenotypes [58,78,80,81]), although another laboratory has had difficulty with this breeding scheme [79]. A second model, which involves breeding commercially available C57BL/6 XY* mice, is more tractable [82]. This model produces XY* gonadal males and XX^Y*^ gonadal males. The X^Y*^ chromosome is a fusion of the non-pseudoautosomal regions of one X and one Y chromosome end to end, connected by an aberrant pseudoautosomal region. Thus, the XX^Y*^ gonadal male is similar to XXY, except that it has only two sex chromosomes. Comparison of XY* and XX^Y*^ indicates that XX^Y*^ have some phenotypes typical of KS men, including hypergonadotropic hypogonadism, infertility due to lack of sperm, Leydig cell hyperplasia, and behavioral deficits [59,82,83]. Therefore, the XY* model has advantages because of its easier breeding scheme and validated features similar to KS. All previous mouse models of KS have a similar disadvantage, in that they produce XXY and XY gonadal males only, so that differences in sex chromosome complement are potentially confounded with lower levels of testicular hormones found in XXY mice [58,59]. In addition, the XX^Y*^ mouse is not completely identical genetically to XXY [48]. Although a few studies on previous mouse models of KS have eliminated XXY vs. XY differences in the levels of testosterone in adulthood by castrating mice and implanting them with testosterone pellets [43,80,81,83], the XXY and XY groups could nevertheless be different because of long lasting (organizational) testosterone effects on phenotypes caused by lower androgen secretion in XXY mice during early critical periods of development, including prenatally. Thus, in mouse models comparing only XXY gonadal males with XY gonadal males, it is difficult to separate organizational hormone effects from the direct effects of sex chromosomes on non-gonadal tissues.

In the SCT model, the gonadal sex of the animal is independent of sex chromosome complement, allowing for improved study of the individual contributions of direct genetic effects, hormonal effects, and interactions of the two. The SCT model has the novel advantage of allowing for the study of the related chromosomal abnormality XYY, which causes a syndrome similar to KS but that does not involve low testosterone levels, and which has not been well studied in animal models. The XYY animals are also a useful control for comparing the effects of an extra sex chromosome to the effects of an extra X chromosome specifically. Similarly, XX males serve as control for two X chromosomes without a third sex chromosome. The main disadvantages of the SCT model are the larger number of genotypes produced and that the model is probably only viable on an outbred background such as MF1.

Some caveats are warranted. It is possible that the expression of *Sry* from the transgene is not identical to that from the endogenous *Sry* from wild-type (WT) mice. Phenotypically, XY^−^(*Sry+*) males are identical to WT XY males in many but not all measured phenotypes [46]. Because *Sry* is expressed outside of the testes, in the brain, kidney, adrenals, and other tissues [84,85], it is not possible to rule out differences between gonadal males in the present study relative to those carrying an endogenous *Sry* gene. Because the treatment of adult mice in the current study was for 3 weeks, it is possible that longer treatments with testosterone would have had more dramatic effects on phenotype.

Although the association between the XXY karyotype and KS was discovered nearly 60 years ago, the mechanisms by which the extra X chromosome causes the syndromic features of KS are still not well understood. Currently, the treatment of KS often involves testosterone replacement therapy, which is understudied but reported to have positive effects on language, intellectual, and motor skills of pre-school KS boys [34,35]. Evidence suggests that in humans, not all KS phenotypes are the result of lower androgen levels [28,31,33]. Because direct sex chromosome effects are difficult to identify in patient populations or in previous animal models, they are poorly understood. The SCT model offers the opportunity to establish which phenotypic features of XXY and XYY are independent of, or dependent on, the gonadal effects of these trisomies. It lays the foundation for using the power of mouse molecular genetics to discover the X genes that cause KS phenotypes and the Y genes that make XYY mice different from XY.

## Conclusions

Using the novel SCT model, we have found that XXY mice have greater body weight and increased adiposity, relative to XY mice. These differences occur in mice that have comparable testosterone levels as adults and are not explained by differences in levels of testicular secretions before adulthood. The SCT model offers promise for further mechanistic understanding of the differences among XXY, XYY, and XY mice that may model differences in humans with similar genotypes.

## Competing interests

The authors declare that they have no competing interests.

## Authors' contributions

XC, SMW-B, TCN, NG, and HB performed the experiments and analyzed the data. SMW-B performed the literature review and drafted the manuscript. RM organized and implemented mouse breeding and genotyping. KR, EV, and APA conceived the experiments, organized the research team, and revised and edited the manuscript. All authors read and approved the final manuscript.

## Supplementary Material

Additional file 1 **Body composition in gonadally intact mice before gonadectomy and after gonadectomy and treatment with testosterone. Figure S1:** body composition in gonadally intact mice before gonadectomy. (A) Gonadal males had about 70% greater absolute fat mass than gonadal females, irrespective of sex chromosome complement (*F*(1,86) = 14.92, *p* = 0.0002, overall ANOVA). (B) In the overall ANOVA, gonadal males had about 21% greater lean mass weight than gonadal females (*F*(1,86) = 79.63, *p < 0.000001), irrespective of sex chromosome complement, and the effect of sex chromosome was significant (*F*(3,86) = 2.83, *p = 0.043). In pairwise comparisons, XXY mice had about 10% more lean mass than XY (*F*(1,51) = 8.88, *p = 0.004). Values are mean ± SEM. **Figure S2:** body composition after gonadectomy and treatment with testosterone. (A) Gonadal males had about 121% greater fat mass weight than gonadal females, irrespective of sex chromosome complement (*F*(1,84) = 34.42, *p < 0.000001). XXY mice had about 60% more body fat mass than XY (*F*(1,51) = 7.08, *p = 0.01). (B) Gonadal males had 17% greater lean mass than gonadal females (*F*(1,84) = 63.71, *p < 0.000001) irrespective of sex chromosome complement. XXY mice had 7% more lean mass than XY (*F*(1,51) = 5.14, *p = 0.028). Values are mean ± SEM. Click here for file
