# Supplementary figures and images for: The Sex Chromosome Trisomy mouse model of XXY and XYY: metabolism and motor performance
Source: Biol Sex Differ. 2013 Aug 8;4:15. doi: 10.1186/2042-6410-4-15 (PMC3751353; doi:10.1186/2042-6410-4-15)

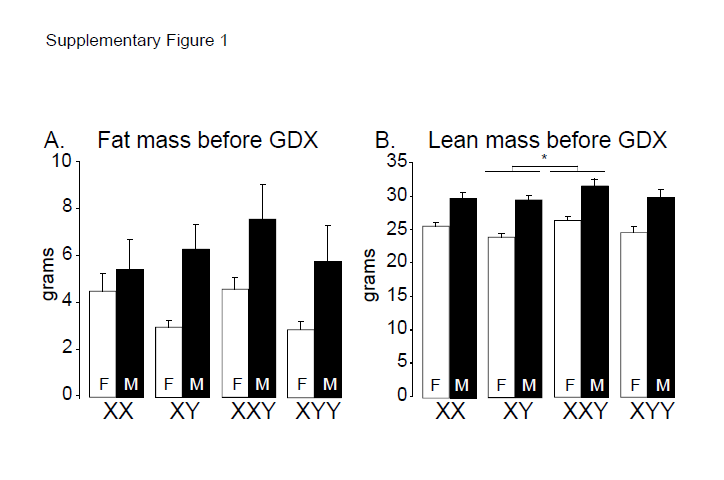


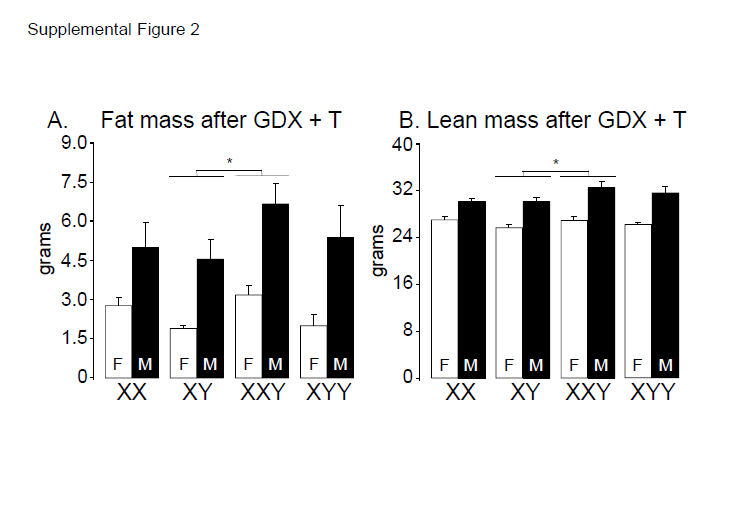

Supplement: Additional file 1 — Body composition in gonadally intact mice before gonadectomy and after gonadectomy and treatment with testosterone. Figure S1: body composition in gonadally intact mice before gonadectomy. (A) Gonadal males had about 70% greater absolute fat mass than gonadal females, irrespective of sex chromosome complement (F(1,86) = 14.92, p = 0.0002, overall ANOVA). (B) In the overall ANOVA, gonadal males had about 21% greater lean mass weight than gonadal females (F(1,86) = 79.63, *p < 0.000001), irrespective of sex chromosome complement, and the effect of sex chromosome was significant (F(3,86) = 2.83, *p = 0.043). In pairwise comparisons, XXY mice had about 10% more lean mass than XY (F(1,51) = 8.88, *p = 0.004). Values are mean ± SEM. Figure S2: body composition after gonadectomy and treatment with testosterone. (A) Gonadal males had about 121% greater fat mass weight than gonadal females, irrespective of sex chromosome complement (F(1,84) = 34.42, *p < 0.000001). XXY mice had about 60% more body fat mass than XY (F(1,51) = 7.08, *p = 0.01). (B) Gonadal males had 17% greater lean mass than gonadal females (F(1,84) = 63.71, *p < 0.000001) irrespective of sex chromosome complement. XXY mice had 7% more lean mass than XY (F(1,51) = 5.14, *p = 0.028). Values are mean ± SEM. [file 2042-6410-4-15-S1.doc]
